# Phenol-stacked carbon nanotubes: A new approach to genomic DNA isolation from plants

**Published:** 2014-09

**Authors:** Farhad Nazarian-Firouzabadi, Ahmad Ismaili, Sayed Mahmoud Zabeti

**Affiliations:** Agronomy and plant breeding group, Faculty of Agriculture, Lorestan University, Khorramabad, Iran

**Keywords:** DNA isolation, Carbon nanotube, Phenol, Plants

## Abstract

Extraction of intact quality DNA from plant tissues, especially those rich in secondary metabolites, is often challenging. Literally, hundreds of different DNA isolation protocols from various plant species have been published over the last decades. Although many commercial DNA isolation kits are convenient and designed to be safe, their cost and availability cause limitations in small molecular labs in many developing countries. In nearly all protocols and DNA isolation kits, phenol and chloroform are used to precipitate various classes of impurities. However, phenol is partially soluble in water, resulting in the co-existence of proteins in upper (aqueous) phases. This phenomenon results in the contamination of the nucleic acids and low quality DNA. Nanotechnology advances have helped many areas of molecular biology such as the development of new diagnosis and purification kits. In this study, for the first time, we report a different approach to isolate DNA from plants based on carbon nanotubes (CNTs). The results show that the phenol reagent stack on CNTs can effectively remove proteins, polysaccharides and other polyphenol constituents. The A260/A280nm absorbance ratios of isolated DNA samples were 1.9 and 1.8 for chamomile and opium plants, respectively, indicating the high purity of the isolated DNA. DNA yield was more than two times the standard Doyle and Doyle method. Furthermore, the isolated DNA proved amenable to PCR ampliﬁcation, using Random Amplified Polymorphic DNA (RAPD) analysis.

## INTRODUCTION

Good quality genomic DNA (gDNA) is of great importance for many downstream applications in molecular biology methods. With the science of DNA recombinant methods growing, the advent of many molecular markers and the importance of genetic diversity studies, the need for the preparation of quality DNA is now becoming a major concern [1, 2]. Without high quality DNA, such downstream molecular manipulations are not feasible [3]. Although several protocols have been described in the literature to isolate quality DNA from plant species, most of them fail to yield high quality genomic DNA. Furthermore, molecular and cell biology laboratories in developing countries suffer from financial problems on the one hand, and a lack of standard facilities to use hazardous chemicals, on the other [4]. One of the major reasons for the failure to obtain high quality genomic DNA is that plants, especially medicinal herbs, produce chemical compounds such as polysaccharides, polyphenols, tannins, alkaloids, flavanoids and terpenes. These compounds interfere with DNA isolation procedures, resulting in low and poor quality genomic DNA [5, 6]. Furthermore, in some instances, reports indicate that commercial kits fail to produce high quality gDNA [7-9]. 

Many wildly used protocols for isolating DNA from a wide range of species were introduced by investigators [10-14]. Although these protocols have been successfully used in their original or modified forms for many plant species, none is accepted and applicable to all plants. As a result, each research group has to establish and modify one of these protocols to obtain quality DNA for a particular need and plant species. Isolating quality DNA from medicinal plants such as opium poppy and chamomile mean struggling with many secondary metabolites. In such circumstances, key modifications required to discard contaminations involve the use of varying concentrations of cetyltrimethylammonium bromide (CTAB), NaCl, polyvinylpyrroli-done (PVP) and other antioxidants [3]. 

Catalysts are chemical substances that accelerate chemical reactions. In most DNA purification methods, phenol is used to precipitate enzymes and proteins released during cell lyses. Since phenol binds to denatured protein molecules and is denser than water (DNA is soluble in water), it accumulates at the bottom of the tubes. One of the major drawbacks of phenol extraction methods is its partial solubility in water. This may lead to low quality DNA because of the co-existence of proteins in the upper (aqueous) phase which contain nucleic acids. To overcome this drawback, chloroform is often used in most DNA isolation methods. Chloroform is immiscible to water; therefore, it not only separates proteins well, but also ensures that the phenol stays organic. 

Recently, several isolation methods have been put forward using a variety of solid-phase supports such as carbon nanotubes. Nucleic acid molecules are bound to the solid supports through hydrogen-bonding and hydrophobic and electrostatic interactions. To date, a number of studies have focused on the development of new solid supports and/or surface modification protocols to enhance the efficiency of isolation and/or recovery of nucleic acid molecules on the carrier surface [15, 16]. In addition, much attention has been paid to the application of nanostructure materials, especially carbon nanotubes (CNTs), which are produced by rolling up single or multiple graphene sheet layers to form concentric cylinders [17, 18] In particular, there have been an increasing number of CNT applications in several fields of chemical analysis including the removal of impurities and pollutants from the environment [19], vaccine delivery [20] and their use as protein transporters [21]. CNTs adsorb phenolic compounds and form nanocatalysts to enhance chemical reactions and remove troublesome organic impurities [22]. Although, Shakhmaeva et al (2011) used multi-layer CNTs to adsorb certain forms of nucleic acids, they did not follow nucleic acid isolation procedures, nor did they compare their results to a standard method. According to their research, only nucleic acids with certain forms could be adsorbed [16]. 

Many medicinal plants such as chamomile *(Matricaria chamomilla*) and opium (*Papaver somniferum*) produce various kinds of secondary metabolites. Chamomile alone contains more than 120 different chemical constituents as secondary metabolites with potential pharmacological activities [23]. To extract nucleic acids (e.g., genomic DNA) from a soup of all kinds of secondary contaminants, we used phenol stacked on CNTs. We also followed the protocol described by Doyle and Doyle (1990) to isolate genomic DNA from fresh leaves as a standard method. In the Doyle and Doyle protocol, chloroform:isoamyl alcohol (24:1) is used to dissolve and bind to the proteins and lipids of cell membranes. This mixture disrupts the bonds holding the cell membranes together, forms complexes with lipids and proteins and causes them to precipitate out of the solution. In our novel method, we managed to isolate nucleic acids from plant tissues, even from those rich in secondary metabolites. The isolation method presented in this study is simple, efficient and rapid and does not require the chloroform:isoamyl alcohol (24:1) step and the hazardous chemicals which are used in most DNA isolation methods. 

## MATERIALS AND METHODS

**Stacking of phenol on carbon nanotubes: **All chemicals were purchased from Sigma-Aldrich (Gillingham, UK) unless otherwise noted. Loading phenol on the CNTs was done by a two-step procedure: first, multilayer CNTs were opened using an acid oxidation method. Second, the phenol molecules were physically attached to the CNTs via non-covalence bonds. 

**Step 1.** ***Opening multilayer CNTs*** **: **CNTs were opened according to Tsang et al. (1994) [24]. Briefly, CNT was milled and two grams of multilayer CNTs with 20-40 nm diameters were placed in a tube containing 30 ml H_2_SO_4_ and 10 ml HNO_3_ (a 3:1 ratio). The mixture was refluxed for 10 h. Nanotubes were then cooled, filtered, and rinsed three times with deionised water. To adjust the pH between 5-6 opened nanotubes were rinsed three times with deionised water and dried at room temperature*. *

**Step 2.** ***Stacking phenol on CNTs: ***About 50 mg opened multilayer CNTs were mixed and crushed with 2.5g monohydrate citric acid. The mixture was placed in a polymerization syringe connected to a vacuum pump, incubated at 120°C for 30 min and stirred using a magnetic stirrer. The reaction was thereafter continued at 140 °C and 160°C for 1 and 1.5 hour, respectively. One milligram from this mixture was dispersed in a 100 ml balloon and diluted with deionised water. This clear liquid was then mixed with 0.1 M phenol in a 1:1 ratio (V/V). Finally, the mixture was exposed to ultrasonic noise three times in 15-minute intervals.

**Characterization of sacked particles: **The size and morphology of magnetic nanoparticles were observed by transmission electron microscopy (TEM, PHILIPS CM120, The Netherlands) operated at 120 kV. FT-IR spectrophotometric analysis was carried out by KBr pellets using a Nicolet 320.

**DNA isolation: **The pre-chilled mortar and pestle were used to ground fresh young leaves (1g) in the presence of liquid nitrogen. Ground leaf materials were added to a 700 µl preheated (60°C) extraction buffer (2% CTAB, 100 mM Tris-HCl, pH 8.0, 20 mM EDTA, pH 8.0, 0.5 M EDTA, pH 8.0 and 1.4 M NaCl). The samples were incubated at 60°C for 30 min and occasionally mixed to avoid aggregation of the homogenate. Afterwards, 400 µl of Phenol, stacked on CNTs, were added to the extract and vortexed thoroughly. The resulting mixture was centrifuged for 5 min at 13000 rpm at 20°C. The upper phase was then transferred to a clean tube. This step was repeated three times to clear the aqueous phase which was added to 0.6 volume of isopropanol by inversion and incubated at -20°C for 15 min to precipitate the nucleic acids. The mixture was centrifuged at 13000 rpm for 15 min (4°C). The supernatant was gently poured off and the pellet was rinsed twice with 70% cold ethanol and centrifuged at 13000 rpm for 10 min (4°C). Finally, the pellet was briefly air-dried and resuspended in a 100 µl 0.5× TE buffer containing 2 mg/ml RNase. The mixture was incubated at 37°C for 30 min, followed by 10 min inactivation at 60°C. 

**Random amplified DNA (RAPD) analysis: **To check the quality of the isolated DNA from the selected medicinal plant leaves, a PCR reaction was performed, using random primers OPH19-5'-TCT CAG CTG G-3' in a DNA Matercycler® (Eppendorf, Hamburg, Germany). Reactions without DNA were also used as negative controls. Each 15 µl reaction volume contained about 40 ng template genomic DNA in a 1× PCR buffer (10 mM Tris-HCl pH 8.3; 50 mM KCl), 3 mM MgCl_2_, 0.2 mM dNTP Mix (Invitrogen), 0.5 µM single primers and 0.2 U Taq DNA polymerase (Invitrogen). The mastercycler was programmed for an initial denaturation step of 5 min at 94°C, followed by 35 cycles in 45 seconds at 94°C, 1 min at 52°C. Extension was carried out at 72°C for 1 min and the final extension at 72°C for 5 min. PCR products were electrophoresed on 1.5% (w/v) agarose gels in a 1×TBE buffer at 100 V for 90 min and stained with ethidium bromide (0.5 µg/ml). Gels with amplification fragments were visualized and photographed under UV light.

## RESULTS AND DISCUSSION

**Figure 1:**
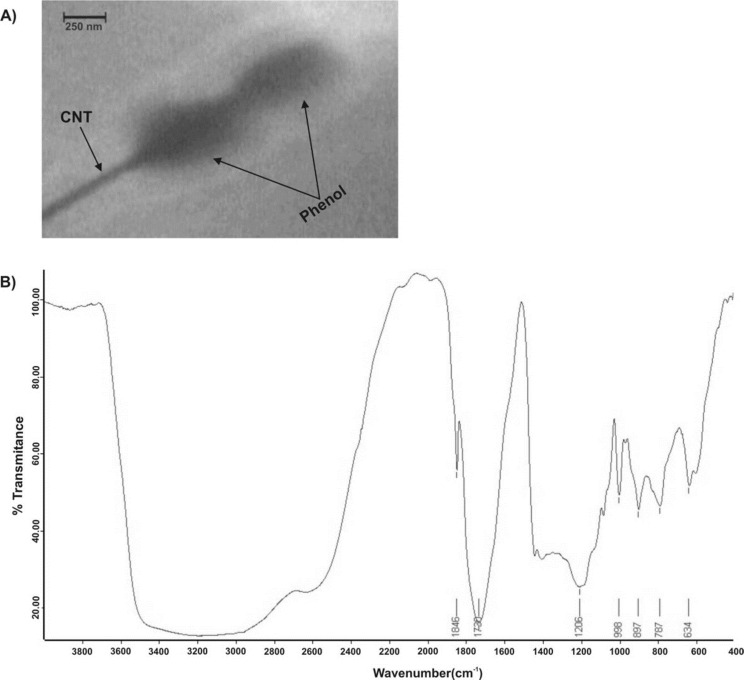
**A** **)** Transmission electron micrographs (TEM) of stacked phenol-CNTs nanoparticles. **B)** FT-IR spectra of the oxidized CNTs with phenol

**Infrared spectroscopy and characterization of grafted CNTs: **Size and morphology of stacked CNT particles were characterized by TEM. A typical TEM micrograph of such particles is shown in Figure 1A. The TEM images showed that phenol was attached to the CNTs. The π-π staking is clearly seen in Figure 1A, indicating that a phenolic ring is in charge of the connection between the CNTs and the phenol molecules. In order to confirm the oxidation of the CNTs and the formation of functional acidic groups, FT-IR measurements were performed. Figure 1B shows the FT-IR spectra of oxidized CNTs. As seen from the figure, the oxidized CNT spectrum shows absorption peaks at 1730cm^−^^1^ corresponding to the C=O stretching vibration from the carboxylic acid (–COOH) groups [25]. A broad peak at approximately 3500cm^−^^1^, which is characteristic of an O–H stretch, was observed due to alcoholic, phenolic or carboxylic groups.

**Figure 2:**
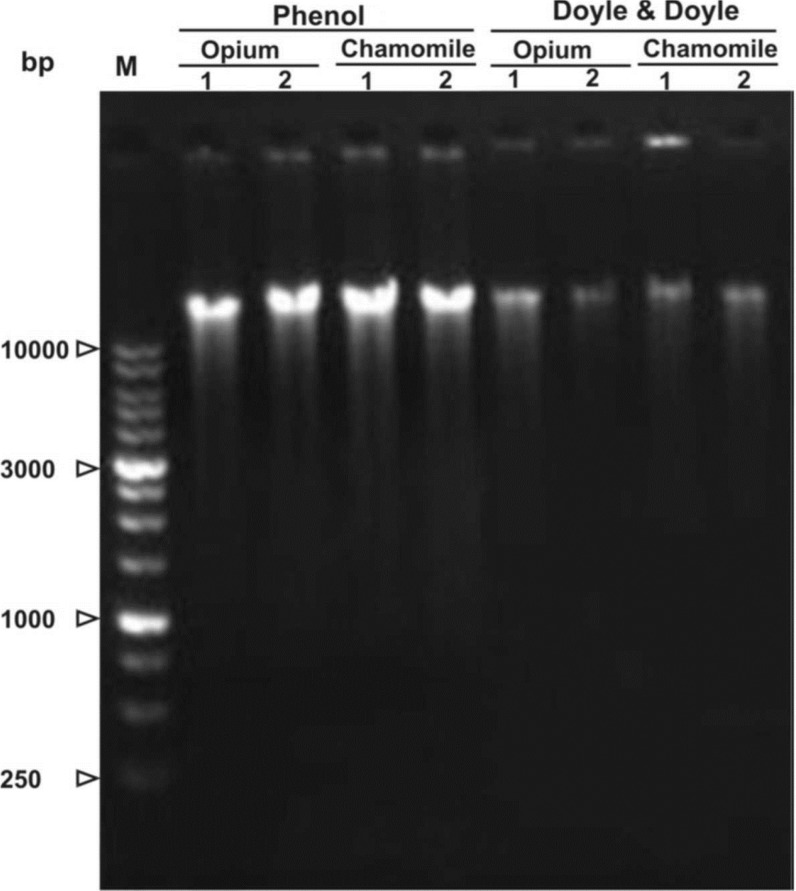
Total genomic DNA extracted by carbon nanotube based phenol, and standard Doyle and Doyle method [10] from opium and chamomile leaves. Numbers indicate two different genotypes of each plant. Lane M, 1Kb DNA ladder marker

**Quantity and quality of isolated nucleic acids: **It is possible to assess DNA concentration by several different methods including absorbance (optical density), agarose gel electrophoresis, fluorescent DNA-binding dyes, etc. Absorbance (measured using a spectrophotometer) and agarose gel electrophoresis analyses are the two most common methods of measuring DNA purity and concentration. As seen in figure 2, using our protocol resulted in much higher and better DNA yields and resolutions from samples of medicinal plants as compared to the standard Doyle and Doyle procedure (Fig. 2). 

Despite factors influencing the accuracy of A_260/280 _ratios [26, 27], optical spectrometer measurements (A_260_ and A_280_) are frequently used to measure nucleic acid concentration. Pure DNA and RNA are expected to have A_260_/A_280 _ratios of 1.8 and 2.0, respectively [27]. Therefore, a ratio of A_260_/A_280_> 1.8 suggests a slight protein contamination in a DNA/RNA sample. In this study, A_260_/A_280 _nm absorbance ratios for chamomile and opium were 1.8 and 1.9, respectively, indicating the high purity of the isolated DNA in comparison to the lower A_260_/A_280 _obtained from the standard protocol (Table 1). As seen in Table 1, the phenol which was grafted on the CNTs resulted in a higher DNA recovery in both plant species in comparison to the standard Doyle and Doyle method. Using our nanotubes, the DNA yield from plant tissues was 944 and 785 µg g^-1^ for chamomile and opium plants, respectively, indicating higher yields in comparison to the basic Doyle and Doyle protocol (Table 1). This confirmed the purity of the DNA and the extent to which it was free of polysaccharide and polyphenol contamination, which could have otherwise, inhibited Taq DNA polymerase activity [1]. Furthermore, gel electrophoresis was consistent with both DNA concentration and A_260_/A_280_ rations. Although the standard Doyle and Doyle protocol always yields high amounts of genomic DNA, the disadvantage of this method is not only the toxicity of phenol/chloroform but also the problems the leftovers might cause with molecular biology enzymes (PCR, digestion, etc).

**Table 1:** DNA yield and the optical spectrometer measurements of opium and chamomile plants are shown, according to utilization of carbon nanotube reagents

**Plant species**	**Method**	**DNA yield (μg g** ^-1^ ** fresh weight** **)**	**A**_260_ **/A**_280_
**Chamomile**	Phenol Doyle & Doyle	944±8.7 381±2.7	1.9±0.14 1.5±0.01
**Opium**	Phenol Doyle & Doyle	785±9.6 270±4.6	1.8±0.14 1.3±0.14

**Note:** Results are the mean of three independent samples (±SD, standard deviation).

**Figure 3:**
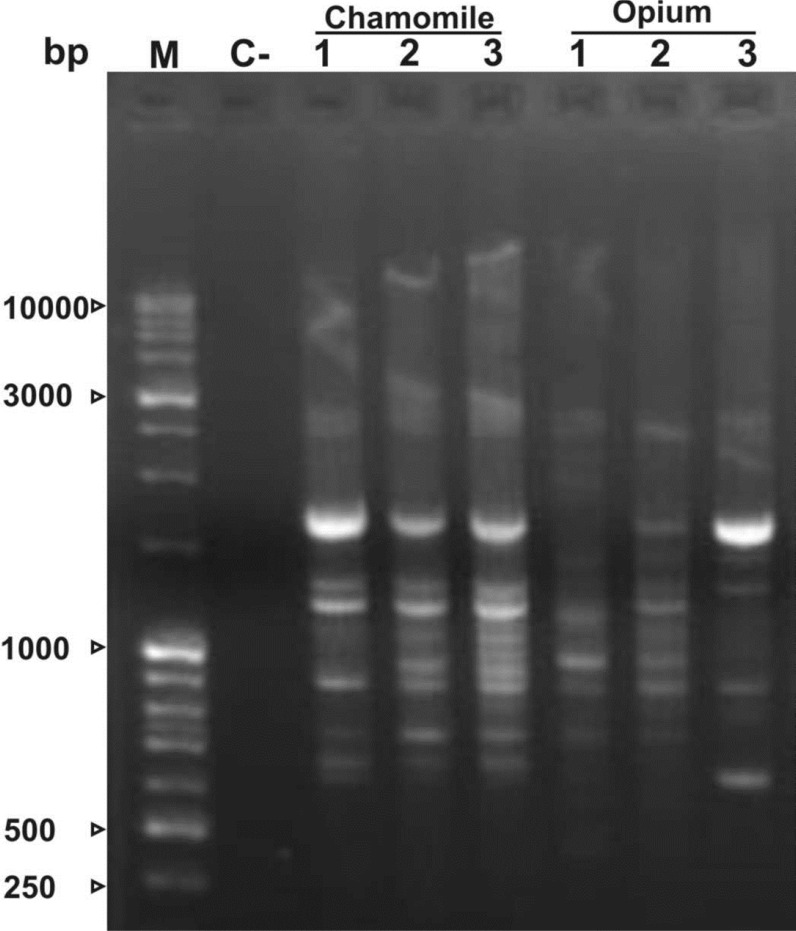
PCR profile of RAPD-PCR amplification products of two medicinal plants genomic DNA (40 ng). Amplification products were fractionated in a 1.5% agarose gel. Lane C- indicates the negative control. Lane M represents molecular marker. Numbers represent different RAPD- PCR products amplified from genomic DNA of three different accessions of each plant using OPH19 (5'-TCTCAGCTGG-3'). Numbers indicate three different plants of each species

In the present study, isolated DNA was used as template in the Rapid Amplified Polymorphic DNA (RAPD) analysis (Fig. 3). The clear amplified DNA bands suggest that the DNA was intact and usable in various downstream molecular applications.

In conclusion, phenol and chloroform are both organic solvents and lyse cell components and the hydrophobic parts (e.g. membrane lipids, hydrophobic protein or polysaccharides, etc) trapped in these solvents. Furthermore, both solvents are powerful protein denaturants and leave behind hydrophobic segments to interact with organic solvents and hydrophilic segments to interact with an aqueous solution. Thus, during organic DNA extraction, interphases (containing proteins or polysaccharides etc.) are seen. Using phenol is advantageous because it separates proteins well, but the drawback is that it is soluble in water, and will contaminate genomic DNA. Since in this method, phenol is attached to the CNTs, it does not stay in the aqueous phase and interfere with DNA downstream applications. In the present study, CNTs were used for the first time as carriers for stacking phenol constituents for genomic DNA purification. We managed to develop a less hazardous protocol for fast and effective nucleic acid extraction by sackingphenol on CNTs. This protocol is far more effective for extracting nucleic acids compared to other basic DNA extraction methods, and does not require *chloroform*:*isoamyl alcohol*.
